# The cost of illness analysis of inflammatory bowel disease

**DOI:** 10.1186/s12876-023-02648-z

**Published:** 2023-01-19

**Authors:** Majid Pakdin, Leila Zarei, Kamran Bagheri Lankarani, Sulmaz Ghahramani

**Affiliations:** Health Policy Research Center, Institute of Health, Shiraz University of Medical Sciences, Shiraz, Iran

**Keywords:** Inflammatory bowel disease, Crohn’s disease, Ulcerative colitis, Cost of illness, Health economics

## Abstract

**Background:**

Inflammatory bowel disease (IBD) is a chronic inflammatory condition involving individuals across all age groups. Recent data suggests the increase in the prevalence of IBD and the surge in applying the biologic drugs in which both change the cost of IBD in recent years. Comprehensive assessment of direct and indirect cost profiles associated with IBD in our area is scarce. This study aimed to determine the economic burden of IBD in Iran from a societal perspective, using cost diaries.

**Methods:**

Patients available on clinic registry and hospital information system (HIS), who were diagnosed with IBD, were invited to take part in this study. Demographic and clinical data, the healthcare resource utilization or cost items, absenteeism for the patients and their caregivers were obtained. The cost of the used resources were derived from national tariffs. The data regarding premature mortality in IBD patients was extracted from HIS. Productivity loss was estimated based on the human capital method. Then, cost date were calculated as mean annual costs per patient.

**Results:**

The cost diaries were obtained from 240 subjects (Ulcerative colitis: n = 168, Crohn’s disease, n = 72). The mean annual costs per patient were 1077 US$ (95% CI 900–1253), and 1608 (95% CI 1256, 1960) for the patients with ulcerative colitis and Crohn’s disease, respectively. Of the total costs, 58% and 63% were in terms of the indirect costs for the patients with ulcerative colitis and Crohn’s disease, respectively. The cost of illness for country was found to be 22,331,079 US$ and 15,183,678 US$ for patients with ulcerative colitis and Crohn’s disease, respectively. Highest nationwide economic burden of IBD was found for patients older than 40 years were estimated to be 8,198,519 US$ and 7,120,891 US$, for ulcerative colitis and Crohn’s disease, respectively.

**Conclusion:**

The medication was found to be the greatest contributor of direct medical costs. Productivity loss in terms of long-term disability and premature mortality were major components of IBD’s economic burden in Iran.

**Supplementary Information:**

The online version contains supplementary material available at 10.1186/s12876-023-02648-z.

## Introduction

Ulcerative colitis (UC) and Crohn’s disease (CD) are included in the spectrum of disorder defined as inflammatory bowel disease (IBD), the relapsing–remitting and chronic inflammatory condition in which the gastrointestinal tract is affected [1]. IBD has lower prevalence compared to other common gastrointestinal-related disorders, such as irritable bowel syndrome, gastroesophageal reflux, and colorectal cancer; however, it is one of the gastrointestinal-related disorders with the most economic burden [1]. Data from many countries, including India [2], China [3], Scotland [4], and Turkey [5] showed an unprecedented growth of IBD worldwide. Concurrently, the incidence of the disease is increasing in Asia [6] and Iran [7]. Low mortality of the disease, the diagnosis at early ages, and its chronic nature have driven this increase in the disease's prevalence. Using biological medications in the treatment of IBD changed the need for hospitalization and surgeries and also changed the cost of the disease in recent years. So, these highlight the importance of the economic burden evaluation of the disease [8, 9]. As stated before, in Iran, the IBD incidence is rising while information on its cost is scarce. Two studies have evaluated direct medical cost and hospitalization cost of the disease [10, 11]. Nevertheless, health policy makers should have reliable information regarding the cost of illness to quantify the impact of the disease on a society. This can inform healthcare cost projection, as well as resource allocation [12]. Regarding the mentioned points, it is necessary to assess the cost of IBD, including direct medical costs, direct non-medical costs, and indirect costs to determine the economic burden of IBD in Iran. In our study, we aim to evaluate the cost of IBD in a multicenter setting.

## Methods

### Participants and data collection

This cost of illness analysis was conducted on the patients diagnosed with IBD in 2021. For data collection, we used the phone records available in Shiraz’ hospital information systems (HIS) and IBD clinic at Faghihi hospital, which is referral IBD clinic affiliated to the Shiraz University of Medical Sciences. Using the convenient sampling method, patients were invited to take part the study through phone calls. Then, the data was obtained through a face-to-face interview while the patients were referred to the clinic. This clinic and referral hospitals all provide the care to the IBD patients, mostly from Fars province and sometimes from neighboring provinces. Our study as a partial economic evaluation technique, aimed to calculate the total costs of IBD in Iran from the society perspective. The IBD diagnosis was confirmed based on clinical, endoscopic, and histological criteria, as described elsewhere [13]. Demographic data (including sex, age, marital status, educational level, educational level, income, and hours of paid work), clinical data (including disease duration, disease progression, and extra-intestinal involvement) were obtained from all participants, and they were interviewed to fill out the cost diary. The disease progression was defined by calculating Mayo score and Crohn’s disease activity index (CDAI) in patients with UC, and CD, respectively. To obtain an estimated prevalence of IBD in Fars province, the population ratio of Fars province in Iran was multiplied by an estimated prevalence of IBD in Iran in 2021 [14, 15], which found to be 1800. The sample volume was determined to be 233 patients for estimation of costs of illness based on 95% CI, margin error of 6%, and the estimated prevalence of IBD in Fars province (http://www.raosoft.com/samplesize.html).

### Cost diary

Cost diary is an instrumental method which is developed by Goosens et al. and is used to estimate the cost of a condition. Since no significant difference has been found among the patients’ report and medical records, there is no need to check medical reports when the cost diary is used [16].

In order to prevent recall bias, the number of physician visits, physiotherapy, purchased drugs dosage, and all other related disease ‘s costs during 3-month prior to the interview were asked from patients, which were scaled up by a factor of four to extrapolate to the mean number of each item during 1-year. Only for hospitalization and surgeries, the duration of 1- year was considered in cost diary. To minimize missing information and partial responses, telephone contacts were made after the initial visit, whenever it was needed. We had a protocol for phone calls. We made phone calls, in case of none- response we considered maximum number of three phone calls in different hours of day and variable days of week to make telephone contacts. Diary records were used to estimate resource used. The cost of the used resources were derived from national tariffs. The costs were recorded in Rial and then we converted to US dollars based on the moving average of the exchange rate in the 2021 252,000:1 (Rial:US) (Additional file 1: Table S1) .

### Death registration database

We extracted death data of patients with IBD, who were died because of IBD-related causes, from HIS during 2013–2021 to estimate economic burden of premature mortality for UCCD.

### Direct medical costs

Direct medical costs included physician visits, physiotherapy sessions, nutrition sessions, purchased medication, surgeries, and para-clinical tests. The cost of each item expected on medication was estimated by the mean number of each item, multiplied by the contemporary tariff of the Ministry of Health and Medical Education (MoHME). The contemporary tariff is defined for each unit of healthcare service item by the MoHME annually in two forms of private and public services. In our study, we used the weighted average of these two forms based on the last national utilization survey [17]. The cost of purchased medication was calculated by the number of each item, multiplied by the unit cost of each item defined by Food and Drug Administration (http://irc.fda.gov.ir/nfi#).

### Direct nonmedical costs

Transportation, self-help group, hours of paid household help, and goods-related to the condition (including alternative medicine, assistive devices, special diet, and books) were considered the components of direct nonmedical costs. Transportation cost was obtained from the sum of public and personal transportation. Public transportation cost was calculated based on the mean number of public transportation service use multiplied by the tariff of transportation defined by Municipal. Personal transportation and other items were calculated based on the real reported costs by patients in the cost diary.

### Indirect costs

The human capital approach was used to estimate indirect costs. Productivity losses because of disability and premature mortality were considered components of indirect costs in our study. We divided disability into short- and long-term disability.

### Productivity losses due to short-term disability

Productivity losses in terms of short-term disability were obtained from temporary absenteeism from work for patients with the disease, and the caregivers. To calculate productivity loss because of temperament absenteeism of patients from work for therapy appointments, the hourly wage of lowest-paid unskilled government workers (LPUGW) of the Ministry of Labor was multiplied by the number of absence hours for each patient. For calculation of the productivity loss because of absenteeism of the caregiver the number of hospitalization days for the patients who were hospitalized during 1-year was added to 14 days per hospitalization. Then, the calculated number was multiplied by the LPUGW. It was assumed that work productivity loss was experienced by caregivers during hospitalization days to fulfill the responsibility of caregiving.

### Productivity losses due to long-term disability

Early retirement of patients, permanent absenteeism from work because of disability, and unpaid household work for caregivers of disabled patients were considered estimating productivity losses due to long-term disability. To estimate the cost of early retirement, the amount of lost pension, compared to the full pension, was calculated for any patient who had early retired. To calculate the cost of permanent absenteeism from work, the annual wage of LPUGW was considered for patients who completely could not work, and were not paid a defined benefit pension. To estimate unpaid household work for caregivers of disabled patients, the number of patients who could not take care of themselves were multiplied by the annual wage of LPUGW because of unpaid permanent caregiver responsibility.

### Premature mortality

Standard expected years of life lost (SEYLL) was used to predict the productivity loss due to premature mortality. We used the following formula to estimate SEYLL [18]:

$${\text{SEYLL}} = {\text{N}} \times {\text{L}}_{{\text{x}}}$$

where N identifies the number of deaths at a certain age, and L_x_ refers to the remaining life-expectancy at the age of death. Based on many arguments and the last update of global health estimates by WHO, we decided not to take into account time discounting and age-weighting [19–22]. We obtained mean number of annual deaths by age, gender, and type of disease. The gender-specific remaining life-expectancy at the age of death was calculated based on the 2020 Iran lifetable from World Health Organization [23], and subsequently SEYLL for each disease was calculated by the sum of the two obtained gender-specific SEYLL for the type of disease. Then, the calculated SEYLL was multiplied by gross domestic production (GDP) per capita for Iran, which is defined by World Bank [24], to estimate the total economic burden of premature mortality for each disease. To obtain the mean cost per patient, the total cost of premature mortality was divided by the estimated prevalence of the disease in Fars Province.

### Statistical analysis

All statistical analyses were performed using SPSS 26.0. Mean, and the standard deviation was used to present continuous variables, while categorical variables were shown as frequency and percentage. Costs were reported as mean costs with 95% CI estimated using non-parametric boodstrap sampling.

## Results

### Demographic characteristics

Among 286 patients who were initially invited, 240 patients accepted to take part. The mean ± Standard deviation (SD) age of participants was 41 ± 13 and 39 ± 14 for UC and CD, respectively. Majority of patients were married, and reside in the cities. The gender was approximately equal between males and females in the both diseases, and UC was predominant disease among participants. Descriptive results based on gender, marital status, employment status, disease type, and other variables are summarized in Table 1.

**Table 1:** Patients’ characteristics

Characteristic	Ulcerative colitis (n = 168)	Crohn’s disease (n = 72)
Age, mean (SD), years	40.64 (12.90)	39.11 (14.04)
Age groups		
0–29	34 [20.2]	19 [26.4]
30–39	55 [32.7]	18 [25.0]
≥ 40	79 [47.0]	35 [48.6]
Sex		
Female	81 [48.2]	30 [41.7]
Male	87 [51.8]	42 [58.3]
Marital status		
Single	36 [21.4]	25 [34.7]
Married	125 [74.4]	43 [59.7]
Widowed	5 [3]	0
Divorced	2 [1.2]	4 [5.6]
Employment status		
Non-employed	81 [48.2]	40 [55.6]
Employed	70 [41.7]	22 [30.6]
Disabled	8 [4.8]	7 [9.7]
Retired	9 [5.4]	3 [4.2]
Educational level		
Illiterate	21 [12.5]	7 [9.7]
Diploma	87 [51.8]	42 [58.3]
Bachelor degree or higher	60 [35.7]	23 [32]
Residence		
City	136 [81.0]	54 [75]
Village	32 [19.0]	18 [25]
Disease duration, years	9.49 (7.98)	7.86 (6.69)
Disease progression		
Continuously active	44 [26.2]	15 [20.8]
Intermittently active	81 [48.2]	41 [56.9]
Inactive	43 [25.6]	16 [22.2]
Extra-intestinal involvement		
Liver involvement	23 [13.7]	13 [18.1]
Oral involvement	38 [22.6]	18 [25.0]
Skin involvement	37 [22.0]	14 [19.4]
Vertebra involvement	12 [7.1]	11 [15.3]
Eye involvement	42 [25.0]	21 [29.2]
Joint involvement	59 [35.1]	30 [41.7]
Lung involvement	5 [3.0]	3 [4.2]
Fistula	4 [2.4]	14 [19.4]

SD, standard deviation

Data are presented as n [%] or mean (SD)

### Direct medical costs

Cost items are categorized in Table 2. Number [%] of participants using resources, and mean annual cost for each category are shown in the table for UC and CD, separately.

**Table 2:** Resource utilization and costs per patient

Cost categories	Participants using resources, n [%]		Mean annual costs in US$ (SD) [% of total costs]	
	UC (n = 168)	CD (n = 72)	UC (n = 168)	CD (n = 72)
Total costs	168 [100.0]	72 [100.0]	1076.89 (1159.14) [100.0]	1608.24 (1497.11) [100.0]
Direct medical cost	157 [93.5]	72 [100.0]	445.52 (691.76) [41.4]	586.96 (629.74) [36.5]
Physician visit	131 [78.0]	68 [94.4]	15.93 (31.52) [1.5]	21.56 (27.34) [1.3]
General physician	12 [7.1]	7 [9.7]	1.24 (7.82) [0.1]	2.16 (9.63) [0.1]
Specialist	14 [8.3]	3 [4.2]	1.60 (11.73) [0.1]	0.38 (2.13) [0.0]
Subspecialist	123 [73.2]	66 [91.7]	12.87 (16.19) [1.2]	18.81 (25.04) [1.2]
Psychiatrist	4 [2.4]	2 [2.8]	0.21 (1.45) [0.0]	0.20 (1.18) [0.0]
Physiotherapy	4 [2.4]	0 [0.0]	0.13 (1.01) [0.0]	0.00 (0.00) [0.0]
Nutritionist consult	0 [0.0]	0 [0.0]	0.00 (00.00) [0.0]	0.00 (0.00) [0.0]
Hospitalization	24 [14.3]	25 [34.7]	64.64 (251.35) [6.0]	161.29 (380.94) [10.0]
Para-clinic	124 [73.8]	62 [86.1]	22.93 (37.64) [2.1]	27.04 (50.00) [1.7]
Laboratory	116 [69.0]	60 [83.3]	7.59 (9.93) [0.7]	10.64 (11.10) [0.7]
Imaging	48 [28.6]	29 [40.3]	15.33 (31.79) [1.4]	16.41 (34.07) [1.0]
Medication	152 [90.5]	69 [95.8]	340.38 (633.18) [31.6]	369.97 (441.22) [23.0]
Surgeries	7 [4.2]	8 [11.1]	1.51 (7.59) [0.1]	7.10 (25.40) [0.4]
Direct nonmedical cost	140 [83.3]	65 [90.3]	8.92 (36.29) [0.8]	4.67 (7.69) [0.3]
Transportation				
Public transportation	62 [39.1]	33 [45.8]	0.42 (0.86) [0.0]	0.56 (1.21) [0.0]
Personal transportation	82 [48.8]	34 [47.2]	4.80 (24.02) [0.4]	3.03 (6.36) [0.2]
Medical related goods (including assistive devices, alternative medicine, special diet, related books)	9 [5.4]	6 [8.3]	2.07 (18.68) [0.2]	0.98 (4.12) [0.1]
Paid household help	1 [0.6]	1 [1.4]	0.09 (1.10) [0.0]	0.10 (0.84) [0.0]
Self-help group	0 [0.0]	0 [0.0]	0.00 (0.00) [0.0]	0.00 (0.00) [0.0]
Indirect costs	58 [34.5]	33 [45.8]	622.46 (808.61) [57.8]	1016.62 (1169.81) [63.2]
Short-term disability	42 [25.0]	30 [41.7]	88.49 (221.46) [8.2]	193.00 (472.27) [12.0]
Temporary absenteeism for patients	22 [13.1]	8 [11.1]	51.62 (175.87) [4.8]	88.11 (378.42) [5.5]
Temporary absenteeism for caregivers	24 [14.3]	25 [34.7]	36.87 (126.52) [3.4]	104.89 (217.58) [6.5]
Long-term disability	23 [13.7]	14 [19.4]	266.50 (745.37) [24.7]	436.40 (980.39) [27.1]
Early retirement	7 [4.2]	12 [2.8]	42.01 (216.60) [3.9]	28.99 (201.20) [1.8]
Permanent absenteeism	14 [8.3]	9 [12.5]	174.60 (580.82) [16.2]	261.91 (697.80) [16.3]
Unpaid household work for caregivers of disabled patients	4 [2.4]	5 [6.9]	49.89 (320.39) [4.6]	145.50 (536.37) [9.0]
Premature death	–	–	276.47 [24.8]	387.22 [24.1]

UC: Ulcerative colitis; CD: Crohn’s disease; SD: standard deviation

Drugs, which were taken by patients, are classified into six categories (Table 3). Number [%] of participants taking each medication’s type, and mean annual cost for each type are shown in the table based on the disease type.

**Table 3:** Frequency and costs of purchased medication

Drug category	Participants taking medication, n [%]		Mean annual costs in US$ (SD) [% of total medication costs]	
	UC (n = 168)	CD (n = 72)	UC (n = 168)	CD (n = 72)
Aminosalicylates	128 [66.2]	46 [63.9]	244.63 (554.37) [71.9]	100.72 (205.08) [25.4]
Biological agents	14 [8.3]	19 [26.4]	74.46 (264.97) [21.9]	235.11 (425.19) [59.3]
Corton	47 [28.0]	24 [33.3]	1.86 (4.23) [0.5]	2.03 (4.36) [0.5]
Immunomodulators	48 [28.6]	35 [48.6]	6.17 (22.09) [1.8]	12.96 (47.79) [3.3]
Supplementary	68 [40.5]	37 [51.4]	5.25 (10.77) [1.5]	7.82 (15.49) [2.0]
Psychotropic drugs	11 [6.5]	9 [12.5]	0.40 (2.05) [0.1]	0.82 (3.89) [0.2]
Miscellaneous	53 [31.5]	25 [34.7]	7.60 (16.92) [2.2]	10.51 (20.19) [2.6]

Miscellaneous drug groups include analgesics, gastrointestinal associated drugs, and others

UC: Ulcerative colitis; CD: Crohn’s disease; SD: standard deviation

Medication had the greatest share of direct medical costs in both types of IBD, accounting for 31.6% and 23.0% of the total costs in the patients with UC and CD, respectively (445.52$ per patient in UC, and 586.96$ per patient in CD). Among types of medication, aminosalicylates contributed to the most prescription proportion in both diseases (66.2% in UC and 63.9% in CD). It shared the highest cost of medication types in patients with UC, accounting for 244.63$ per patient. Less than twenty percent of patients consumed biological agent; however, this type of medication accounted for the highest and the second highest medication costs in patients with CD (235.11$), and UC (100.72$), respectively (Table 3). Hospitalization was another important contributor of direct medical costs, especially in the patients with CD, responsible for 10% of the total costs (161.29$) per patient. Physiotherapy and nutrition consult almost did not impose cost to the studied IBD patients (Table 2).

### Direct nonmedical costs

Although the proportion of using nonmedical resources were high by participants, the related costs accounted for less than 1% of the total costs in both diseases (8.92$ per patient in UC, and 4.67$ per patient in CD). They were mostly driven by transportation (Table 2).

### Indirect costs

Indirect costs were major contributors of the total costs, responsible for more than a half of the costs (622.46$ per patient in UC, and 1016.62$ per patient in CD). Productivity losses induced by short-term disability were more frequent among participants, as compared with the losses due to long-term disability, and were almost equally formed by two components of temporary absenteeism for patients and caregivers. Despite the lower frequency of productivity losses due to long-term disability (13.7% in UC patients, and 19.4% CD patients), they imposed a large share of the total costs (266.50$ per patient in UC, and 436.40$ per patient in CD). Permanent absenteeism from work was predominant compartment of productivity losses induced by long-term disability in both disease types (174.60$ per patient in UC, and 261.91$ per patient in CD). Productivity loss because of premature death was another important contributor of the total costs, accounting for almost a quarter of the costs in both diseases (276.47$ per patient in UC, and 387.22$ per patient in CD) (Table 2).

### Cost of illness for country

The characteristics of participants, including age, sex, disease type and age at diagnosis were almost equivalent to those described for the recent pilot feasibility study of first nation-wide IBD registry in Iran, which enabled us to extrapolate the mean annual total costs regarding disease type and age group for the country [17]. An estimated prevalence of IBD in Iran in 2021 was used for the calculation [15]. The cost of illness for country was found to be 22,331,079 and 15,183,678 for UC and CD, respectively (Table 4).

**Table 4:** Mean annual total costs in US$ for country

Age group (year)	Ulcerative colitis	Crohn’s disease
0–29	6,454,537	2,422,262
30–39	7,678,023	5,640,525
≥ 40	8,198,519	7,120,891
All age groups	22,331,079	15,183,678

## Discussion

In this retrospective cohort study, we aimed to determine the economic burden of IBD, and to compare the profiles of costs in UC and CD patients. There are barriers to study the economic burden of specific diseases in Iran, as diagnostic data is not ordinarily coded for outpatient visits, and the patients’ resource utilization are not recorded. Thus, there is the paucity of information on resource utilization consumed by patients with specific diseases. However, to the best of our knowledge this study is among the first comprehensive studies in Iran assessing both direct and indirect cost profiles associated with IBD.

Several studies have evaluated the economic burden of IBD in other countries [25–37]. Consistent with their findings, we found that the CD patients’ resource utilization was higher than that of UC patients, and this difference was more considerable in hospitalization, surgeries, and laboratory sectors. Variations in the pathogenesis of the two diseases can explain this difference, as UC might not lead to irreversible and systemic damage observed in CD patients [38]. However, the amount of difference among the annual mean costs of UC and CD differs from a study to another, based on the design and population of study, as inconsistencies are created in the results of cost of illness studies when comparing one to another due variations in method [30]. Annual mean costs per patient for UC and CD were between 6217–11,477 US$, and 11,034–18,932 US$ in the USA, respectively. The corresponding ranges were 8949–10,395 euros, and 2898–6742 euros in European countries [39, 40]. In our study, annual mean costs per patient for UC and CD were found to be 1077 US$ (95% CI 900–1253), and 1068 (95% CI 1256, 1960) respectively, which is in line with the results of studies in UK and Germany in terms of CD: UC costs ratio [25, 34]. These two studies have applied a similar method of using patient-reported resource utilization to estimate economic burden of the disease (bottom-up approach). The difference of total costs per patient between Iran and European countries could be explained by the fluctuations in Iran’s currency exchange rate to dollar in the last few years.

According to our findings, the medication use represents the major source of direct medical costs (76.4% and 63.0% of direct medical costs in UC and CD patients), while costs related to surgery and hospitalization have not a large share of direct cost. The results of more recent studies are consistent with our results [30–33]. The widespread application of biological agents in the treatment of IBD patients has changed the healthcare outlook, leading to a substantial shift in cost profiles [33]. The effect of this type of medication was significant on the reduction of colectomy in UC patients [41–43]. According to findings, despite the relatively low proportion of patients who received biological agents (8.3% and 26.4% of UC and CD patients); they shaped a significant contributor of the total costs in both diseases. This highlights the importance of better insurance coverage for such drugs to make them affordable. According to results, none of the participants used the nutritional consultation and assessment. Considering the importance of supportive nutritional therapy in the clinical care of IBD patients [44, 45], routine assessment and monitoring of nutritional status in IBD patients in Iran is highly encouraged.

Direct nonmedical costs were minor contributors to the total costs, as compared with direct medical and indirect costs. Based on our findings, none of participants had been a member of self-help groups. In these groups, patients share their own experiences, which can lead to applying executable strategies for management of chronic disease by other patients [46]. Therefore, it is pivotal to design self-help groups for IBD patients in Iran which fit for their need; subsequently, they should be encouraged to enroll in such groups.

Short- and long-term productivity losses because of disability were also evaluated. We found that productivity losses because of disability handled 33.0% and 39.1% of the total costs in UC and CD, respectively. According to a German study, long term productivity losses shared 32% and 49% of the total costs in UC and CD, respectively [34]. In a more recent study in the Netherlands, productivity losses due to IBD-related absenteeism were found to be 16% and 39% of the total costs, respectively [33]. It seems that biological agents have had an effective role in reducing productivity losses because of disability in CD patients. However, due to different methodologies in measurement of productivity losses due to disability, we have limitations for a more detailed comparison with the results of other studies. In our study, we considered absenteeism and unpaid household work for caregivers, which were important contributors to costs for CD patients, accounting for 6.5% and 9.0% of the total costs, respectively. We did not evaluate presenteeism in our study, since no validated questionnaire is available to evaluate presenteeism with a recall time of over 7 days [33].

The premature mortality was another major contributor of costs in both diseases, which is in line with findings of systematic analysis of the global burden of inflammatory bowel disease. It shows the fact that IBD-associated premature mortality forms a great share of disease burden in countries with low socio-demographic index (SDI) [47]. There is insufficient population-based data evaluating the causes of premature mortality in IBD patients in Iran. In a study assessing the trend of colectomy in the country, colorectal cancer was found to be the leading cause of death in IBD patients undergoing colectomy. In this regard, cancer screening protocols should be routinely performed in IBD patients [9]. Cancer and cardiovascular disease were found to be leading causes of mortality in IBD patients in the USA. Therefore, healthy lifestyle behaviors should be routinely assessed in IBD patients, and adherence to such lifestyle should be encouraged to reduce contributing risk for cancer and cardiovascular disease, and subsequently the risk of premature mortality and related costs in IBD patients [48].

### Limitations

This study provided a more comprehensive view of the costs of illness for IBD in the Iran. However, this study was limited by the small sample size and prevalence approach for cost diaries. Furthermore some aspects of estimation might be affected by purchasing power of participants.

## Conclusion

The medication was found to be the greatest contributor to direct medical costs. Productivity loss due to long-term disability and premature mortality were major components of inflammatory bowel disease burden in Iran.

## Supplementary Information

**Additional file 1: Table S1.** Unit Costs of Medical Resources Consumed by Patients/*: For Nurition Consult, Imaging, Laboratory, and Surgery constant of k should be multipled by given data; k = 13600.

## Data Availability

The data that support the findings of this study are available from the corresponding author upon reasonable request.
